# Transcriptional Changes Fade Prior to Long-Term Memory for Sensitization of the *Aplysia* Siphon-Withdrawal Reflex

**DOI:** 10.1523/ENEURO.0477-25.2026

**Published:** 2026-03-27

**Authors:** Tania Rosiles, Melissa Nguyen, Robert J. Calin-Jageman, Irina E. Calin-Jageman

**Affiliations:** Neuroscience Program, Dominican University, River Forest, Illinois 60305

**Keywords:** forgetting, gene expression, long-term memory, sensitization, transcription

## Abstract

Forming a long-term memory requires changes in neuronal transcription. What happens, though, as the memory is forgotten? And how does the transcriptional state relate to the maintenance and recall of the long-term memory? To answer these questions, we have been systematically tracing the time course of transcriptional changes evoked by long-term sensitization in the marine mollusk *Aplysia californica*. Our approach captures transcriptional changes in neurons of known behavioral relevance using a within-subject design, delineating patterns of transcriptional change that are comprehensive and reproducible. We have previously reported that within 1 d of long-term sensitization training, there is a widespread transcriptional response involving robust changes in over 5% of tested transcripts (1,198 of ∼22 k; Conte et al., 2017). Within 1 week, however, memory strength fades, and nearly all transcriptional changes relapse to baseline ( Perez et al., 2018). Here we report microarray analysis (*N* = 16) of transcriptional changes 5 d postlearning, a time point when memory strength has weakened but is still robust. Remarkably, we found that at this intermediate behavioral stage, nearly all transcriptional changes have fully decayed, even in subsets of animals that have shown very little forgetting. Thus, most transcriptional changes seem to decay more rapidly than memory expression. We discuss several possible ways that memory expression could become decoupled from detectable transcriptional regulation.

## Significance Statement

This project characterizes the transcriptional state accompanying a partially forgotten long-term memory in *Aplysia*, showing that most transcriptional changes induced during learning fade before forgetting is complete. These results raise interesting questions about the interrelationships between transcriptional, neuronal, and behavioral change.

## Introduction

Long-term memories are distinguished not only by their duration but also by their requirement for changes in neuronal gene expression (Goelet et al., 1986). After a memory is induced, though, how do transcriptional states relate to memory expression? The “consensus model” is that transcriptional changes trigger the encoding of long-term memory (Klann and Sweatt, 2008) and that this includes activation of self-sustaining biochemical changes that perpetuate at least some transcriptional changes to support the ongoing expression of the memory (Zhang et al., 2010; Smolen et al., 2019). With this model, forgetting is a disruption of maintenance mechanisms, and transcriptional states and memory expression should be tightly linked. There is some evidence, though, that the relationship between transcription and memory expression is not straightforward. For example, some transcriptional changes seem to persist even after memory expression weakens (Kim et al., 2012; Perez et al., 2018), and savings memory (the rapid relearning of a seemingly forgotten memory) can be expressed without notable changes in gene expression (Rosiles et al., 2020). A critical goal, then, is better characterizing the ways in which changes in neuronal gene expression sculpt not only the induction but also the maintenance and forgetting of long-term memory.

**Figure 1.**
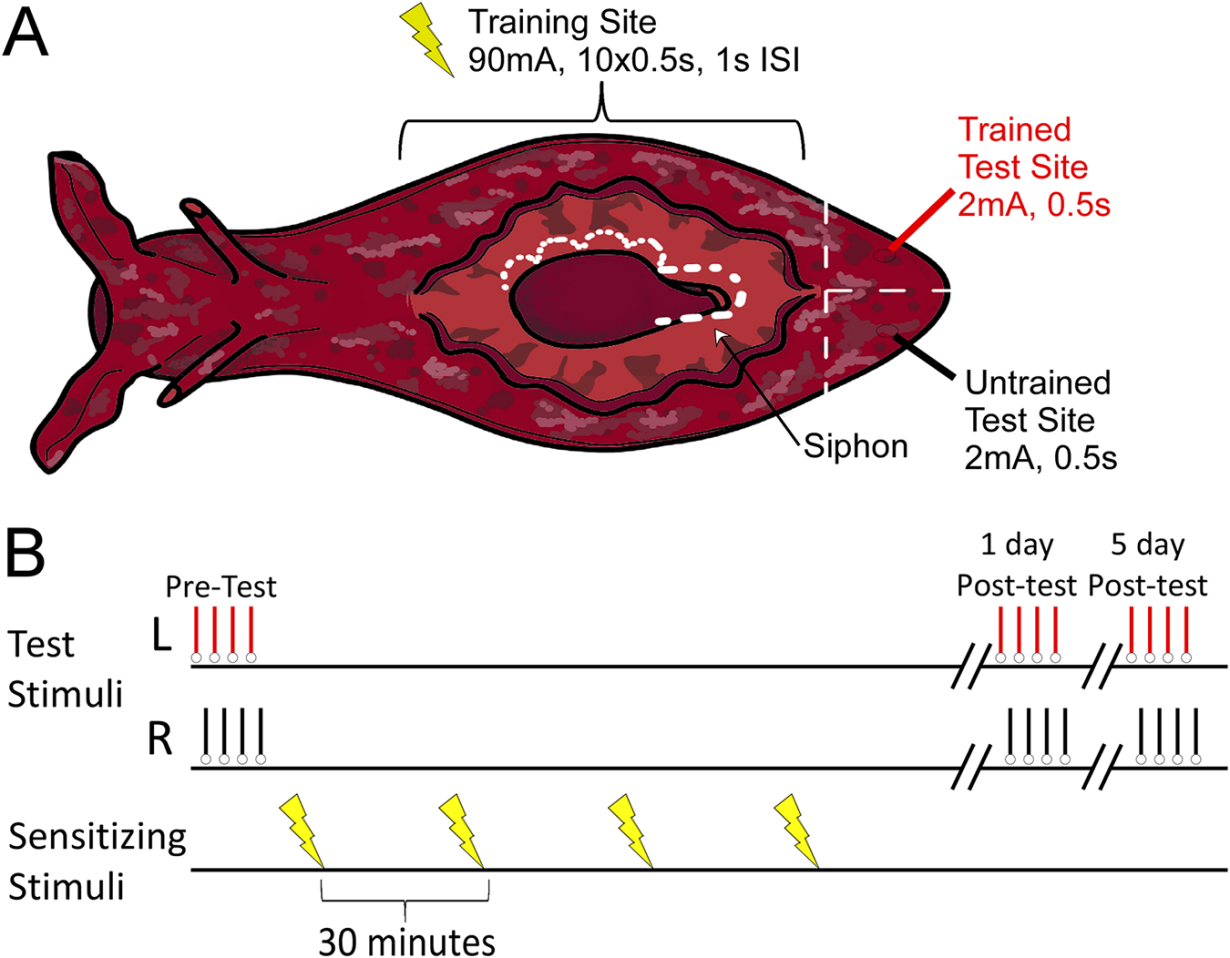
Long-term sensitization of the T-SWR. ***A***, A diagram of the body of an *Aplysia*. T-SWRs are evoked by applying an innocuous shock to the left or right tail (black and red lines). The duration of the T-SWR serves as an index of behavioral responsiveness. For LTS training, a noxious shock is applied along the length of one side of the body (lightning bolts). ***B***, Experimental protocol. First, baseline T-SWR measures are made on the left and right side of the tail; then LTS training is applied to the one side of the body; then T-SWR measures are made again 1 and 5 d after posttests. Immediately after the 5 d posttests, pleural ganglia from the trained and untrained side are harvested in matched pairs of left-trained and right-trained animals.

We have been studying this issue by tracing the changes in gene expression that accompany long-term sensitization of the tail-elicited siphon–withdrawal reflex (T-SWR) in *Aplysia californica* (Fig. 1*A*). Sensitization is an evolutionarily conserved form of memory (Walters, 2018) in which a painful stimulus causes an increase in reflex responsiveness, often accompanied by additional behavioral changes and modulated by the current motivational state of the animal (Shields-Johnson et al., 2012; Leod et al., 2018; Mueller et al., 2025). In *Aplysia*, long-term sensitization can be induced through repeated noxious shocks applied to the one side of the body (Scholz and Byrne, 1987; Wainwright et al., 2002) and then observed as a long-term, unilateral increase in the duration of the T-SWR, a defensive reflex (Walters and Erickson, 1986). The circuitry mediating this reflex has been characterized, and long-term sensitization has been shown to be due, in part, to long-term increases in the excitability and synaptic strength of the VC nociceptors located in the pleural ganglia (Scholz and Byrne, 1987; Walters et al., 2004). This form of learning has proven especially useful for studying transcriptional correlates of long-term memory because (1) the unilateral expression of sensitization enables powerful within-subject comparisons of gene expression and (2) analysis of gene expression can proceed from the pleural ganglia which contain the VC nociceptors, providing an excellent transcriptional signal from neurons of known behavioral relevance to the induction and maintenance of sensitization memory (Calin-Jageman et al., 2024).

We have previously shown that long-term sensitization training produces widespread changes in gene expression in the pleural ganglia containing VC nociceptors (as well as directly in the VC neurons themselves). One hour after training, there is notable upregulation of ∼80 transcripts, many of which are transcription factors (Herdegen et al., 2014a,b). At 1 d after training, this transcriptional response ramifies, involving over 1,100 transcripts that show clear regulation (Conte et al., 2017). After 1 week, the expression of sensitization fades completely (reflex responsiveness returns to baseline), and so do most transcriptional changes, with the vast majority of transcripts regulated 1 d after training showing a statistically significant decline in regulation (Perez et al., 2018). Although most transcriptional changes decay within 1 week, we have identified seven transcripts which show “beyond forgetting” regulation that persists for up to 2 weeks, long after recall of sensitization has fully decayed (Patel et al., 2018; Perez et al., 2018). Interestingly, sensitization memory can be rapidly relearned (savings or latent memory; Philips et al., 2006) and can then repersist in a long-term form (for >1 d), but this does not reactivate transcriptional changes (Rosiles et al., 2020), providing a puzzling disconnect between transcriptional states and memory expression.

Here we characterize the transcriptional changes occurring 5 d after long-term sensitization training, a time point at which there is substantial forgetting and yet still clear expression of long-term sensitization memory. Following a preregistered design, we designed our study to help answer four questions:

1. What is the fate of the ∼1,200 transcripts strongly regulated during early maintenance of long-terms sensitization?
2. Is the small set of “beyond forgetting” transcripts also regulated at this earlier time point?
3. Does the passage of time and forgetting of sensitization produce additional, late-breaking transcriptional changes?
4. Do any gene expression changes predict individual levels of forgetting?

## Materials and Methods

Prior to conducting microarray analysis, we preregistered our sample size plan, data exclusions, all manipulation and measures, and a complete microarray analysis script (https://osf.io/g3ueu). We then followed our preregistered plan exactly, with no notable deviations. We report below all preregistered analyses and note as exploratory any follow-up analyses developed after data collection. Our preregistration documents, analysis scripts, and behavioral data are posted to the Open Science Framework (https://osf.io/ccwks/); the microarray data are also posted to NCBI's Gene Expression Omnibus (Geo ID: GSE315391).

### Animals

Animals (75–125 g) were obtained from the RSMAS National Resource for *Aplysia* and maintained at 16°C in one of the two 90 gallon aquariums with continuously circulating artificial seawater (Instant Ocean, Aquarium Systems). Handling was as described previously (Herdegen et al., 2014b). *Aplysia* are true hermaphrodites.

### Long-term sensitization training

A 1 d long-term sensitization training protocol (Fig. 1*B*) was used, adapted from Wainwright et al. (2002) but with a stronger shock (90 vs 60 mA) and a constant-current stimulus (Bonnick et al., 2012; Cyriac et al., 2013). Training consisted of four rounds of noxious shock applied at 30 min intervals to one side of the body with a handheld electrode. Each round of shock consisted of 10 pulses (60 Hz biphasic) of 500 ms duration at a rate of 1 Hz and an amplitude of 90 mA. During the course of each shock, the stimulating electrode was slowly moved from anterior (just behind neck) to posterior (just in front of tail) and back to cover nearly the entire surface of that side of the body. The side of training was counterbalanced.

### Behavioral measurement

As a behavioral outcome, we measured the duration of the T-SWR (Walters and Erickson, 1986). The reflex was evoked by applying a weak shock to the one side of the tail using a handheld stimulator (60 Hz biphasic DC pulse for 500 ms at 2 mA of constant current). T-SWR behavior was measured as the duration of withdrawal from the moment of stimulation to the first sign of siphon relaxation. Behavioral measurements were made by a researcher blind to the side of training.

To track sensitization memory, we measured T-SWR durations before training (baseline) and then 1 and 5 d after long-term sensitization training (posttests).

We focused on changes in T-SWR behavior by calculating the log-fold change from baseline to posttest at each time point on both the trained and untrained side of each animal:

$$\mathrm{LFC}=\mathrm{Lo}g_{2}(\mathrm{Post}\text{-}\mathrm{Test}/\mathrm{Baseline}).$$

By this metric, a score of 0 represents no change in T-SWR duration, and scores above 0 represent sensitization. We then calculated the degree of sensitization expression at each time point by comparing the change in T-SWR behavior on the trained side to the untrained side:

$$\mathrm{Sensitization}\_\mathrm{Expression}=\mathrm{LF}C_{\mathrm{Trained}}-\mathrm{LF}C_{\mathrm{Untrained}}.$$

Finally, we calculated a forgetting index as the change in sensitization expression from Day 1 to Day 5:

$$\mathrm{Forgetting}\_\mathrm{Score}=\mathrm{Sensitization}\_\mathrm{Expressio}n_{\mathrm{Day}1}\text{–}\mathrm{Sensitization}\_\mathrm{Expressio}n_{\mathrm{Day}5}.$$

With this metric, a score of 0 occurs when memory expression is stable (same expression on Day 1 and Day 5 leads to a forgetting score of 0) and is above 0 (indexing forgetting) when memory expression is lower on Day 5 than on Day 1.

### Isolation and processing of pleural ganglia RNA

We compared gene expression in the pleural ganglia on the trained versus untrained sides. To control for lateralized gene expression, samples from two animals trained on opposite sides were pooled (*N* = 32 animals) to create 16 sets of trained versus control microarray samples.

To analyze transcription, pleural ganglia RNA was isolated immediately after the 5 d posttest. These ganglia contain the VC nociceptors which help mediate the expression of long-term sensitization memory through training-induced long–lasting increases in excitability and synaptic efficacy (Cleary et al., 1998). Isolation and homogenization were exactly as described in Herdegen et al. (2014b).

### Sample size determination

We set a target of 16 microarray samples (requiring 32 animals). Our previous work has shown good sensitivity with eight microarray samples; we doubled this target to account for the fact that regulation 5 d after training might grow more subtle than observed 1 d after training. Our sample size target exceeds the consensus recommendation of at least five biological replicates per group (Pavlidis et al., 2003; Tsai et al., 2003; Allison et al., 2006).

### Quality controls

To ensure suitable samples, several quality controls were utilized to select animals for microarray analysis:

1. Animals had to exhibit strong learning, defined as at least a 30% increase in T-SWR duration from baseline to the 1 d posttest.
2. The expression of sensitization had to be unilateral, with less than a 30% increase in T-SWR on the untrained side from baseline to the 1 d posttest.
3. There could be no protocol errors in training, testing, or isolation that would yield ambiguity about the processed samples (e.g., a training shock applied to wrong side).

We ran animals in batches of 12–30. We completed training of 77 animals. Of these, 7 were excluded for exhibiting a weak training response, 6 were excluded for bilateral expression of sensitization, and 32 were excluded due to protocol errors (3 due to a single shock applied to the wrong side during training; 29 due to poor or uneven RNA isolation). This left 32 animals which were paired into 16 samples for microarray analysis. To pair animals for a microarray sample, we first sorted qualifying animals within a batch by the forgetting score and then combined two animals with similar forgetting scores but opposite sides of training (the left-trained animal with the highest forgetting score was paired with the right-trained animal with the highest forgetting score and so on).

### Microarray processing

We used the Aplysia Tellabs Array (ATA; GEO: GPL18666) to characterize changes in gene expression due to long-term sensitization training. This array includes 26,149 distinct probes representing all known sources of *Aplysia californica* ESTs and mRNAs at the time of design (January 2012). Based on estimates from previous microarray designs (Moroz et al., 2006), the ATA should cover >50–60% of all transcripts expressed in the nervous system. Full details on the array design are reported in Herdegen et al. (2014b).

Microarray processing was completed by MOgene. A two-color approach was used with each array hybridized to a sample from a trained or untrained animal. In half of cases, trained samples were hybridized with Cy3 and controls with Cy5; in the other half, we dye-swapped. Processing was exactly as described in Herdegen et al. (2014b).

We identify microarray probes by the NCBI accession number for the EST or mRNA that the probe was designed to. For EST-based probes, when there is a definitive match of the EST to a gene model in the current *Aplysia* genome, we also provide that accession number.

### Statistical analysis

Behavioral responses were averaged by time point. Paired comparisons were made from baseline to posttest for each side. The standardized effect size estimates (Cohen's *d*) are corrected for bias (Hedges, 1981) and calculated so that positive values represent an increase in response (Calin-Jageman and Cumming, 2019, 2024).

Microarray data were analyzed using limma (Smyth, 2005; Ritchie et al., 2015) from the Bioconductor suite of tools (Gentleman et al., 2004) for R (Ihaka and Gentleman, 1996). Our processing script for identifying differentially regulated transcripts was preregistered and is posted on the Open Science Framework. Median expression values were analyzed (Zahurak et al., 2007). These were corrected for background using the normexp + offset algorithm recommended for Agilent microarrays by Ritchie et al. (2007). An offset of 30 was selected based on inspection of MA Plots. Expression was then normalized using the loess function (Smyth and Speed, 2003). Where multiple probes were used to measure the same EST or mRNA, these were averaged. Finally, trained and control expression were compared using an empirical Bayes-moderated *t* test (Smyth, 2004). Statistical significance was calculated using Benjamini–Hochberg correction for multiple comparisons to maintain a 5% overall false-discovery rate (Benjamini and Hochberg, 1995). We used the treat function from limma (McCarthy and Smyth, 2009) to conduct a stringent test for significant regulation. Specifically, rather than using a null hypothesis of no regulation, we tested for regulation that was statistically distinguishable from at least a 10% change in expression in either direction. We have previously found that using this type of high-stringency criterion yields very strong predictive validity in independent qPCR (Holmes et al., 2014; Herdegen et al., 2014b). We also quantified the degree of relationship between the regulation observed 5 d after LTS training with our previous screens of regulation observed 1 d after LTS training (Herdegen et al., 2014b) and 7 d after LTS training (Perez et al., 2018). We examined the correlation between transcriptional states after correcting expression scores for potential measurement error using the genuine association of gene expression profiles function (genas) in limma (Ritchie et al., 2015). Finally, we conducted exploratory analysis of the completion of each gene list using the propTrueNull function (Ritchie et al., 2015) and the convex decreasing densities approach developed by Langaas et al. (2005).

## Results

### Long-term sensitization training produces robust unilateral sensitization that is partly forgotten within 5 d

**Figure 2.**
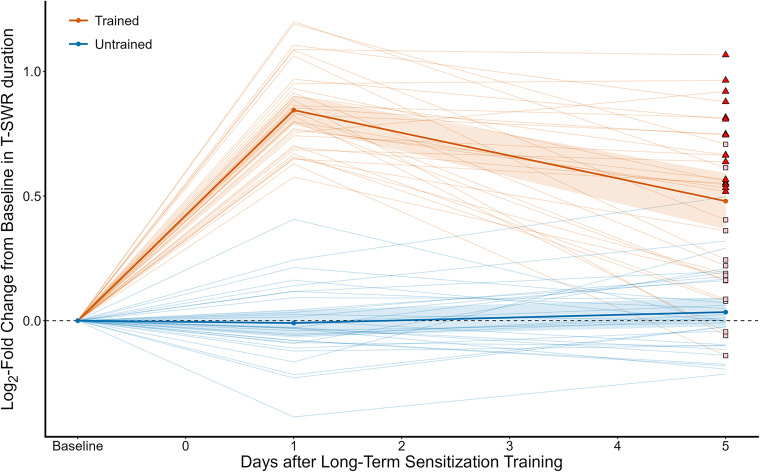
Training produces a long-term sensitization memory that is partly forgotten at 5 d. This figure shows T-SWR duration as the log-fold change from baseline on both the trained (red) and untrained (blue) sides. Dark lines with dots represent group means; shading indicates 95% CI of the mean. Individual animals are represented by the light lines, with a symbol for the 5 d posttest indicating if it was grouped into the low-forgetting group (red triangles) or high-forgetting group (pink squares) for an exploratory analysis to determine if there was more prominent gene expression changes in the low-forgetting animals.

All animals (*N* = 32 qualified animals) received long-term sensitization training (Fig. 1*B*). This produced a robust sensitization memory on the side of training (Fig. 2), with responses increasing from an average of 11.1 s at baseline to an average of 20 s at the 1 d posttest, nearly a doubling in response time (LFC_Trained_ = 0.84 95% CI [0.78, 0.91]). As expected, sensitization was unilateral, as on the untrained side, response durations were at 11.2 s at baseline and 11.1 s at the 1 d posttest, a negligible change in response (LFC_Untrained_ = 0.0 95% CI [−0.06, 0.04]). Thus, sensitization memory was strongly expressed 1 d after training (Sensitization_Expression_day1_ = LFC_Trained_ − LFC_Untrained_ = 0.85 95% CI [0.76, 0.94]; *d*_avg_ = 5.2 95% CI [4.2, 6.5]).

Five days after training sensitization was substantially reduced but still clearly expressed. On the trained side, responses declined to a mean of 16.0 s at the 5 d posttest, only a 50% increase from baseline rather than the doubling observed 1 d after training (LFC_Trained_ = 0.48 95% CI [0.36, 0.60]). As expected, responses on the untrained side were stable, with a mean of 11.4 s, very similar to baseline measures (LFC_Untrained_ = 0.03 95% CI [−0.02, 0.09]). Thus, there was still substantial expression of sensitization on Day 5 (Sensitization_Expression_day5_ = 0.45 95% CI [0.30, 0.59]; *d*_avg_ = 1.7 95% CI [1.1, 2.4]) but with notable forgetting relative to Day 1 (Forgetting_Score = Sensitization_Expression_day1_ − Sensitization_Expression_day5_ = 0.41 95% CI [0.29, 0.53]; *d*_avg_ = 1.2 95% CI [0.8, 1.6]). The mean forgetting score represents a loss of about one-half of the initial expression of sensitization, but there was considerable diversity in forgetting scores, with some animals showing almost no change in expression and others showing essentially no remaining sensitization.

Overall, our protocol succeeded in capturing a partially forgotten state of sensitization where there is clear and yet notably weaker expression of sensitization, with considerable diversity between animals. To capture this diversity, we matched each left-trained animal with a right-trained animal with a similar forgetting score, and we used the average forgetting score for each microarray sample in several analyses reported below.

### To what extent is regulation 1 d after sensitization preserved 5 d after training?

How similar is gene regulation 5 d after training to the pattern observed 1 d after training? To answer this question, we compared changes in gene expression across these time points, focusing specifically on 1,198 transcripts which we have previously identified as strongly regulated 1 d after training (Conte et al., 2017). For this analysis, we did not include a set of transcripts that show very long-lasting regulation; these are analyzed in the next section.

**Figure 3.**
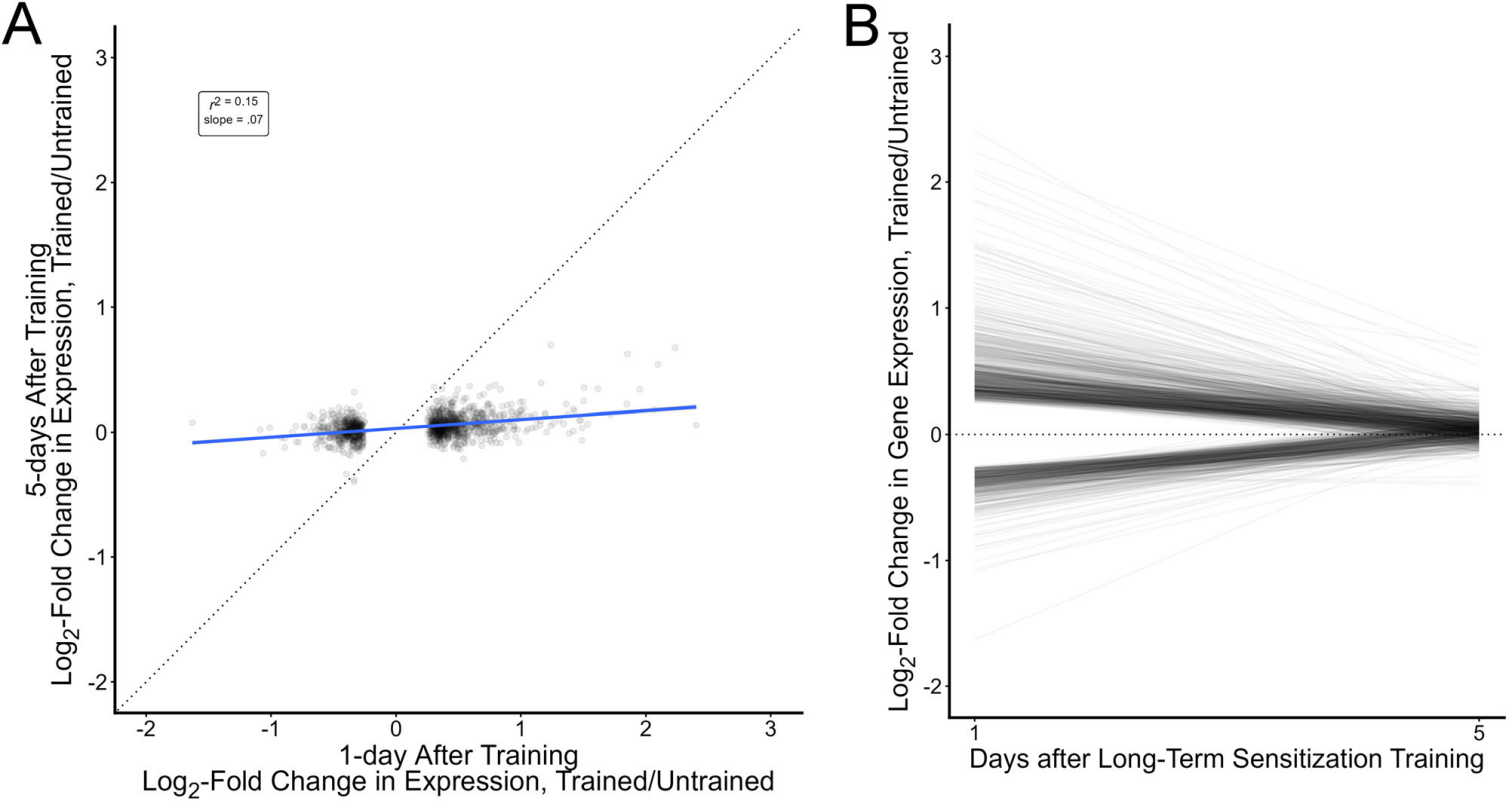
Gene expression changes observed 1 d after training have mostly decayed by 5 d after training. ***A***, Scatterplot of log-fold changes in gene expression (trained vs untrained) 1 and 5 d after long-term sensitization for each of the 1,198 transcripts significantly regulated 1 d after training. The dashed line has a slope of 1 indicating the relationship expected if regulation was perfectly preserved over time. The blue line represents the actual line of best fit (the 95% confidence interval of the line is shaded but too narrow to be visible). The large difference between the unit line and actual slope indicates a substantial attenuation of learning-induced gene expression changes. ***B***, The same data but with each line representing regulation from Day 1 (starting point) to Day 5 (ending point) for a transcript. The dashed line at 0 indicates no regulation. The 1 d data in this figure are from Conte et al. (2017).

Surprisingly, we found that regulation at Day 5 is only modestly correlated with regulation at Day 1 (*r* = 0.38 95% CI [0.33, 0.43]; *r*_corrected_ = 0.54; *N* = 1,198; Fig. 3*A*). Moreover, the slope of the relationship was weak (*B* = 0.07 95% CI [0.06, 0.08]), indicating that, on average, regulation 5 d after training is <10% as strong as what was observed 1 d after training. For comparison, we have previously conducted the same analysis (Rosiles et al., 2020) on two independent samples both collected 1 d after training and found a very strong correlation in expression patterns (*r* = 0.95 95% CI [0.93, 0.96]; *r*_corrected_ = 0.99).

Consistent with the correlational analysis, when we tested for significant regulation in the current dataset, we found that none of the 1,198 transcripts regulated 1 d after training still showed clear regulation 5 d after training (none showed a statistically significant difference between trained and untrained expression against a null of 10% or less change in expression). With 16 paired samples and a focused analysis, this is probably not due to a lack of power. Indeed, analysis of the distribution of *p* values suggested a false-positive rate of 0.0001, giving an expected value of 0 false negatives over these 1,198 comparisons. Figure 3*B* shows the time course for each of these transcripts, with a line representing regulation at Day 1 (Conte et al., 2017) to Day 5 (current experiment); the sharp decline toward no regulation notable in nearly all transcripts indicates the substantial attenuation of regulation that has occurred as time has elapsed since training.

We followed up these preregistered analyses with three exploratory analyses. First, we directly compared regulation at Days 1 and 5. Consistent with the correlational analysis, this showed that 99% (1,181) of the transcripts previously identified as regulated at Day 1 showed a statistically significant decline in regulation at Day 5; this suggests that the lack of regulated transcripts is not due simply to poor power but to a statistically reliable decline in regulation for most transcripts. We also explored using less-stringent criteria for identifying regulated genes (a null of 0 rather than of at least 10%). This increased the number of transcripts qualifying as regulated from 0 to 39 (output from this exploratory analysis is posted to this project's Open Science repository: https://osf.io/ccwks/files/z43ua), still a very small proportion (3%) of the transcripts which had been clearly regulated 1 d after training. Finally, we isolated our analysis to only the samples of animals which had shown low levels of forgetting. Specifically, we split the 16 microarray samples into a “high-forgetting” set and a “low-forgetting” set (Fig. 2; the triangle and square symbols denote the animals in these constructed groups). As expected for intentionally dividing the samples in this way, this produced an enormous difference in forgetting scores across these post hoc groups (*M*_High_forgetting_ = 0.67 95% CI [0.56, 0.79]; *M*_Low_forgetting_ = 0.14 95% CI [0.07, 0.22]; *d*_avg_ = 2.8; 95% CI [1.8, 3.9]), with the low-forgetting group having lost, on average, only 17% of their initial expression of sensitization. We then tested for significant regulation of expression only in the low-forgetting group. This did not substantially alter conclusions: the correlation with Day 1 regulation was actually slightly weaker among the low-forgetting samples (*r* = 0.35 95% CI [0.30, 0.40]; *r*_corrected_ = 0.50; *N* = 1,198), and the number of transcripts passing a stringent test for regulation remained at 0.

Overall, these results suggest that most of the gene regulation apparent 1 d after training substantially weakens within 5 d, though some of the pattern of regulation persists, and at low stringency, a small number of transcriptional changes are detectable. This was true even in animals which did not show substantial forgetting of sensitization.

### To what extent are transcripts regulated for 7 d similarly regulated at 5 d?

**Figure 4.**
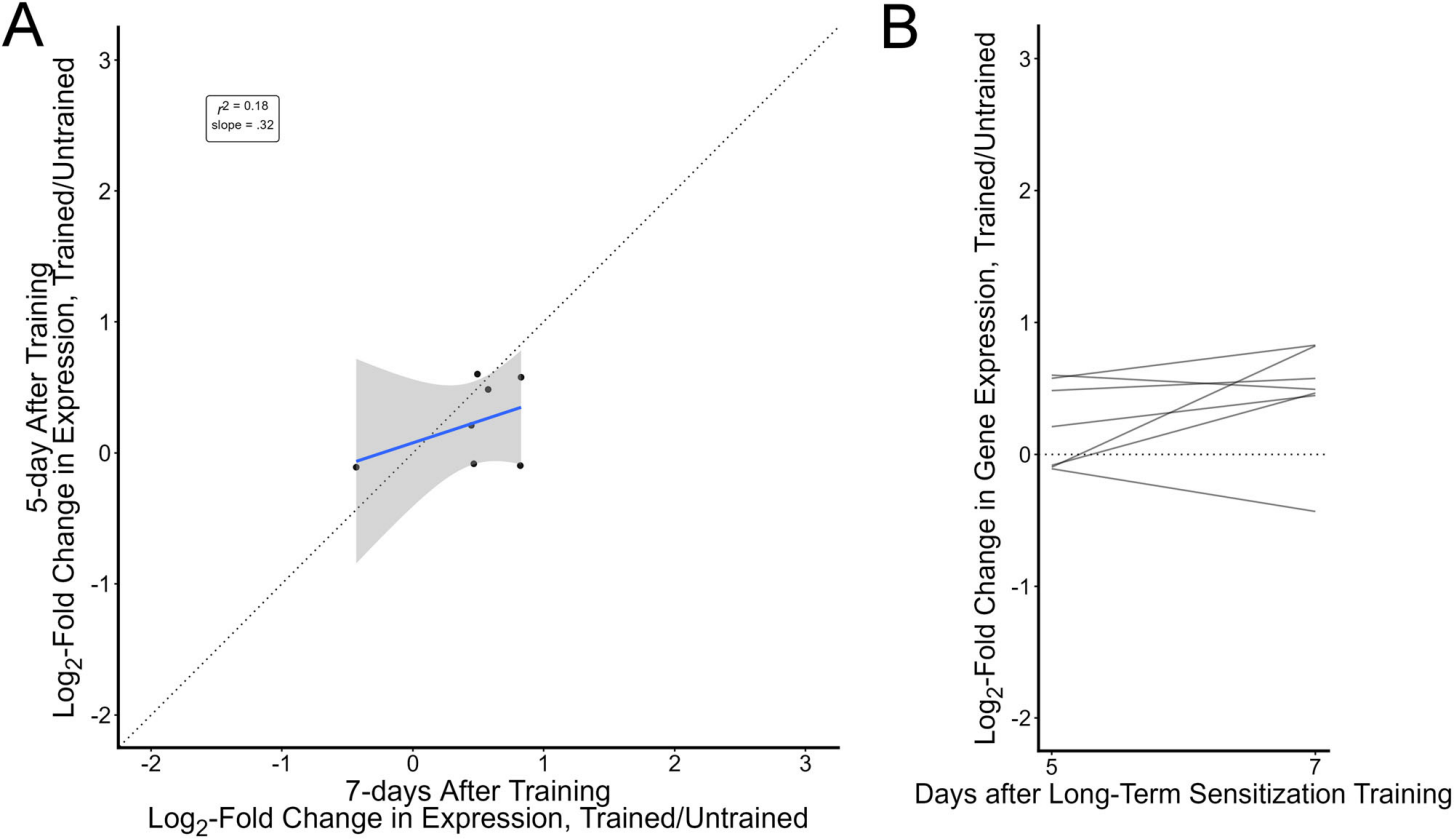
The small core of transcripts regulated 7 d after training are mostly detectable 5 d after training. ***A***, Scatterplot of log-fold changes in gene expression (trained vs untrained) 5 and 7 d after long-term sensitization data for the seven transcripts significantly regulated 7 d after training. The dashed line has a slope of 1 indicating the relationship expected if regulation was perfectly preserved over time. The blue line represents the actual line of best fit and its 95% confidence interval (shading). ***B***, The same data but with each line representing regulation from Day 5 (starting point) to Day 7 (ending point) for a transcript. The dashed line at 0 indicates no regulation. The 7 d data in this figure is from Perez et al. (2018).

We have previously shown that a small set of seven transcripts remain regulated after forgetting, with some showing clear learning-associated changes in expression for up to 2 weeks after training (Patel et al., 2018; Perez et al., 2018). We next examined the extent to which these transcripts are regulated 5 d after training (Fig. 4).

We found that four of these highly persistent regulated transcripts also showed clear regulation at this earlier time point (AH005259.2 FRMFamide precursor; FF066943.1/XM_013087893.2; EB254334.1; EB257711.1/XM_013081020.2). In an exploratory analysis with a less-stringent test, this increased to five (EB255259.1/XM_035971949.1 spectrin). Two transcripts expected to be upregulated were instead very slightly downregulated in this sample (EB243511.1/XM_005100516.3 ankyrin repeat and BTB/POZ domain-containing protein 1; EB342172.1). Overall, levels of regulation in these transcripts were correlated with what we had observed 1 week after training (*r* = 0.42 95% CI [−0.48, 0.89]; *r*_corrected_ = 0.71; *N* = 7; Fig. 4*A*), though the small sample size makes this relationship highly uncertain. Given that we have previously demonstrated that these transcripts are very persistently regulated after training, these results were not surprising. These analyses demonstrate, though, that the study design and approach are sensitive enough to detect a high proportion (five of seven) of regulated transcripts.

### To what extent are new transcriptional changes induced during forgetting?

We next examined if the passage of time might activate additional transcriptional changes that we have not yet observed as related to sensitization, analyzing the 24,839 microarray probes not included in the previous two analyses. After correction for multiple comparisons, no transcripts were flagged as regulated. Although this is a large number of tests for 16 paired samples, the lack of detected regulation did not seem due to low power, as the estimated false-positive rate was 0 at this stringency level.

We followed up this preregistered analysis with an exploratory analysis using a lower level of stringency (null of 0). This identified 47 transcripts which could potentially be late-regulated (output from this exploratory analysis is posted to this project's Open Science repository: https://osf.io/ccwks/files/xzvrf). Of these, 22 showed a statistically significant difference in regulation from what had been observed at Day 1. Even at this low level of stringency, the estimated false-positive rate remained at 0. Thus, at low stringency, there is modest evidence for some late-breaking transcriptional changes, but there is a high risk of flagging what may be negligible levels of regulation.

### To what extent are gene expression changes related to forgetting?

We examined if changes in gene expression might predict forgetting, assessing the correlation between gene expression and forgetting scores across the entire microarray. After correction for multiple corrections, we found no transcripts showing a statistically significant correlation with forgetting scores.

As an exploratory analysis we increased power by restricting the analysis just to the 1,259 “Day 1” transcripts (fewer comparisons requires less aggressive correction, increasing power). This restricted analysis, however, also failed to identify any transcripts significantly correlated with forgetting scores. Similarly, testing the set of seven persistently regulated transcripts also failed to identify any transcripts that showed a significant association with forgetting scores.

A sample size of 16 is small for a correlational analysis, especially with correction for multiple comparisons. However, the main issue was likely the lack of gene regulation observable at this time point (restriction of range).

## Discussion

We examined the transcriptional correlates of a partly forgotten memory. We found that transcription decays more rapidly than behavior, with very little of the pattern of transcriptional regulation observed 1 d after training preserved 5 d after training; this was true even in a subset of animals which had shown very little forgetting. We did not detect any late-breaking transcriptional changes as sensitization memory was forgotten, nor was it possible to predict levels of forgetting from the state of single transcripts. We reconfirmed that a small set of transcripts remains persistently regulated after sensitization training.

**Figure 5.**
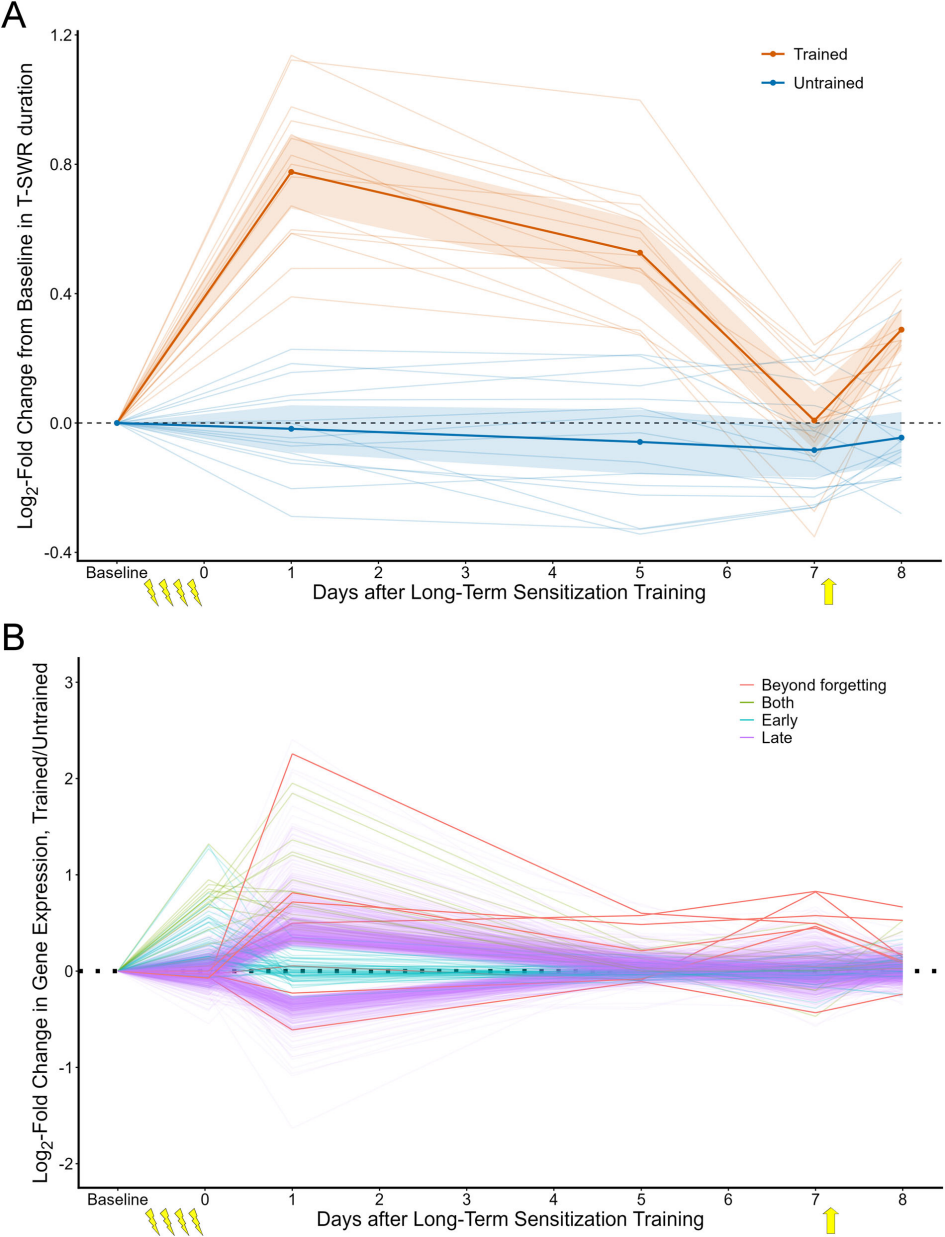
Comparison of the behavioral and transcriptional changes inducted by long-term sensitization training. ***A***, The behavioral time course of long-term sensitization (replotted from Perez et al., 2018). Animals were given long-term sensitization training (lightning bolts) and received a weak reminder shock (yellow arrow) after 7 d posttests, revealing a latent sensitization memory. The plot shows T-SWR duration as the log-fold change from baseline on both the trained (red) and untrained (blue) sides. ***B***, The transcriptional time course of long-term sensitization. This plot summarizes gene expression measured by microarray 1 h (Herdegen et al., 2014b), 1 d (Conte et al., 2017), 5 d (current study), 7 d (Perez et al., 2018), and 8 d but with a reminder shock applied after the 7 d posttests (Rosiles et al., 2020). In total, 64 transcripts show clear regulation at 1 h but not subsequently (“early,” marked in teal); 1,237 show clear regulation at 1 d but not subsequently (“late,” marked in purple), 17 are regulated at both 1 h and 1 d but not subsequently (“both,” brown), and 7 show regulation that at 1 and 7 d (“beyond forgetting,” red). Note that most transcriptional changes decay well before memory expression and that the return of memory expression after a reminder occurs in the absence of detectable changes in gene expression.

This study completes a series conducted to trace the transcriptional correlates of sensitization memory as sensitization is encoded (Herdegen et al., 2014b), maintained (Conte et al., 2017), forgotten (Perez et al., 2018), and then reactivated as a savings memory (Rosiles et al., 2020). Figure 5 integrates our findings from this paper with our previous work. In addition to characterizing the early and late waves of transcription produced by learning, our results show that transcription and memory expression become dissociated after training: behavior can show strong expression of the memory with almost no detectable transcriptional changes, and the few transcriptional changes that persist do so relatively stably even as behavioral expression changes.

Our survey of transcriptional changes after sensitization suffers from several technical limitations. First, it is conducted with whole ganglia, blending together transcriptional changes across multiple cell types, perhaps even canceling out important but offsetting changes in gene expression. We have found that changes in gene expression in whole ganglia correlate strongly with those measured specifically in the VC nociceptors that help encode sensitization (*r* = 0.79; Conte et al., 2017), but the lack of cell-type resolution remains a limitation of our approach. Our survey is also incomplete, as our microarray was developed from an early EST library in *Aplysia* and does not represent all neuronally expressed transcripts. We have recently completed long-read sequencing that will allow the design of a more comprehensive microarray platform. We note, though, that our current design has high coverage and is likely representative of the neuronal transcriptome and thus should provide an accurate global sense of how transcription is regulated by sensitization training. A third key limitation to consider is our use of unilateral sensitization, where we characterize changes in gene expression relative to the untrained side of each set of paired animals. This within-subject design offers high precision, but it is predicated on the assumption that the untrained side does not exhibit major changes in gene expression due to training. This assumption is at least somewhat tenuous; others have reported that with more extensive sensitization, training neuronal correlates become bilateral, with noted outgrowth of VC nociceptors on both the trained and untrained sides (Wainwright et al., 2004). We mitigate this limitation by screening out any animals that show behavioral changes on the untrained side, but we cannot rule out the possibility that our analysis misses transcripts which are bilaterally regulated by training. Thus, it is probably best to think of this work as capturing the transcripts distinctly regulated by memory “expression rather than all changes produced by the training protocol. While each of these limitations deserve consideration, none are likely to fully explain the lack of detectable changes in gene expression we report here 5 d after training, especially given the abundant changes we were able to detect using the same methods and smaller samples sizes at earlier time points. That is, it seems unlikely that there is substantial ongoing regulation restricted exclusively to the transcripts missing from our microarray or that substantive ongoing changes were canceled out by late-breaking changes on the untrained side identical enough to normalize training-induced differences on the trained side yet different enough to not produce the same behavioral consequences. Thus, although the technical limitations of our approach are notable, they do not substantially undermine our conclusion that the expression of sensitization memory becomes decoupled from ongoing transcriptional changes, at least at the level of whole ganglia.

The disconnect between transcription and memory expression is somewhat surprising. Specifically, sensitization has been proposed to activate self-sustaining transcriptional loops that then contribute to maintaining the expression of sensitization memory (Smolen et al., 2019). For example, cellular analogs of sensitization training induce CREB1 phosphorylation, increasing the binding of CREB1 to its own promotor, producing a long-term increase in the expression of CREB1 mRNA and protein (Mohamed et al., 2005; Liu et al., 2011b). This long-term increase in CREB1 transcription is essential for the long-term maintenance of cellular and synaptic correlates of long-term sensitization (Liu et al., 2011a). Moreover, there is evidence that epigenetic changes are required to support the ongoing expression of sensitization (Pearce et al., 2017), and epigenetic marks are generally thought to influence neuronal phenotypes through altered gene expression. Thus, recent reviews (Smolen et al., 2019) have posited that the maintenance of sensitization is at least partly due to ongoing transcriptional regulation. Our findings, in contrast, suggest that the vast majority of transcriptional changes induced after learning are transient and that the few that do persist are not clearly linked to memory expression. What might explain this discrepancy between the hypothesis of transcription-mediated maintenance and the apparent discordance between transcription and memory expression?

One possibility is that we observe an early decay of transcription simply because there is a lag between the decay of transcription and the completion of forgetting, as the proteins produced by transcriptional regulation could extend behavioral expression for some time after gene expression returns to baseline conditions. While the notion of a lag between transcription and behavior makes sense, it would still be unclear why there are so few detectable transcription changes during savings memory (Rosiles et al., 2020). Nevertheless, we are now exploring more intensive training protocols that produce longer-lasting sensitization; this should provide a clearer test for maintenance-related transcription that is not muddied by dynamic behavioral expression.

**Figure 6.**
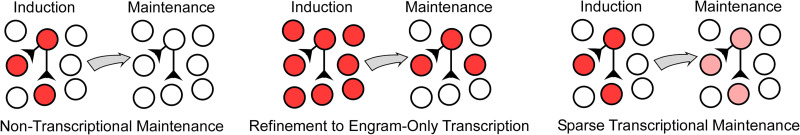
Mechanisms that would yield lower global changes in gene expression during memory maintenance. Left, One possibility is that memory maintenance does not require ongoing transcription, perhaps relying instead exclusively on posttranscriptional mechanisms. Middle, A second possibility is that the initial transcriptional response to sensitization is overly broad and is then refined only to neurons that will encode the memory. Right, A third possibility is that encoding neurons have a broad initial transcriptional response but require only a small subset of ongoing transcriptional changes to maintain the altered signaling that expresses sensitization.

Another possibility (Fig. 6, left) is that maintenance of sensitization is nontranscriptional. For example, cellular analogs of sensitization are accompanied by self-perpetuating conformational change in synaptically expressed CPEB, a prion-like protein that regulates local translation (Si et al., 2003, 2010; Miniaci et al., 2008). This process is essential to early maintenance of synaptic correlates of long-term sensitization. Similarly, cellular analogs of sensitization also produce persistent changes in posttranslational processing of PKC (yielding PKM Apl III), and putative inhibitors of PKM have been reported to eliminate the expression of long-term sensitization in intact animals (Cai et al., 2011). Thus, there are demonstrated maintenance mechanisms that operate solely on the protein level, and our data could be an indication these are the only key factors for sustaining the expression of sensitization. We cannot rule out this possibility, but we note (1) that this makes it hard to understand why the initial transcriptional response is so broad and (2) long-term sensitization is not exclusively encoded via synaptic changes, long-term changes in excitability also contribute, and this at least suggests a cell-wide maintenance mechanism.

Another possibility is that the decline in transcriptional regulation we observe at the level of whole ganglia is due to a process of refinement. For example, the transcriptional response to sensitization training might be initially exuberant and then refined over time to just the subset of neurons that will encode the memory (Fig. 6, center). Refinement could even alter the loci of memory maintenance, perhaps shifting from the VC nociceptors to interneuron or motor neuron populations, leading to negative results in the pleural ganglia we analyzed but with ongoing transcriptional changes in the pedal ganglia and/or peripheral nervous system. We have not observed substantial long-term changes in gene expression in the pedal ganglia after sensitization training (Herdegen et al., 2014b), but cellular analogs of sensitization require biochemical signaling in motor neurons (Villareal et al., 2009) and are accompanied by long-term changes in gene expression (Hu et al., 2015).

In addition to being refined across cell types, it may be that the transcriptional changes required for maintenance are sparse, needing only a few ongoing changes after sensitization is consolidated (Fig. 6, right). This would make sense in terms of the cellular demands of sustaining a memory, as it seems implausible that long-term sensitization requires the ongoing regulation of hundreds of transcripts.

Refinement and sparseness are not mutually exclusive; either of these scenarios would produce the ganglia-wide decline in transcriptional regulation we have observed, but this would belie continued transcriptional maintenance that has become harder to detect. We are now conducting screens with cell-type specificity, which should help clarify if we have missed ongoing but more-specific transcriptional changes.

Overall, the relationships between transcriptional, neuronal, and behavioral change remain surprisingly murky. While long-term memories require changes in neuronal gene expression at the time of learning, it is still difficult to provide a clear account of what, exactly, these diverse transcriptional changes accomplish, how long they last, and how/why forgetting can occur. Resolving these key issues will likely require time course studies that can resolve transcription at the single-cell level with sufficient biological replicates to provide strong precision of measurement.

## Synthesis

Reviewing Editor: Rishikesh Narayanan, Indian Institute of Science Decisions are customarily a result of the Reviewing Editor and the peer reviewers coming together and discussing their recommendations until a consensus is reached. When revisions are invited, a fact-based synthesis statement explaining their decision and outlining what is needed to prepare a revision will be listed below. The following reviewer(s) agreed to reveal their identity: John Byrne, Wayne Sossin.

**Synthesis**

The study addresses the critical question on the relationship between gene transcription and memory retention using Aplysia as the model organism. The experimental design is rigorous, involving powerful within-subjects controls and high-stringency statistical analyses. The primary finding of the study is that most transcriptional changes decay more rapidly than memory expression. These observations imply that robust gene transcription might not be essential for maintenance of memory. Both reviewers agree on the rigor associated with the experimental design as well as on the importance of this study in unraveling the mechanistic basis of memory storage.

However, both reviewers underscore the need to place the findings of the study in context and acknowledge possible caveats within an expanded discussion section. In an expanded discussion section, please elaborate on possible alternate explanations for the findings here, covering both methodological and conceptual aspects. Please respond to all points raised by both reviewers, with appropriate changes made in the revised manuscript addressing their comments.

**Reviewer's comments**

**Reviewer #1**

The manuscript presents a well-designed microarray analysis of transcriptional changes associated with long-term sensitization in Aplysia. The study finds that transcription fully decays at an intermediate behavioral stage (5 days post-sensitization training), a time-point when memory strength has weakened but is still maintained. The conclusion is that most transcriptional changes decay more rapidly than memory expression. This is an extremely interesting finding, suggesting that robust gene transcription may not be needed for the maintenance of memory. The study used a powerful within-subjects design, used unilateral sensitization in Aplysia to compare trained and untrained sides within the same animal, and used high-stringency statistical analyses. The manuscript could be improved by addressing the following concerns.

1. A novel finding is that memory expression persists at day 5, whereas nearly all transcriptional changes return to baseline. The authors need to discuss in more depth possible alternative mechanisms for this negative finding. For example, the negative results could be due, in part, to the use of whole ganglia for the transcriptional analysis. The use of whole ganglia might not capture some changes in expression, because changes from the specific subset of neurons actually encoding the memory may be diluted. The authors briefly discussed this possibility as a "refinement" process. They should provide additional discussion of this methodological limitation and implications of using whole ganglia for the analyses.

2. Another possible explanation for the negative results is the approach of comparing gene expressions in the pleural ganglia collected from the trained vs. untrained sides in the same animal. The authors should discuss the possibility that behavioral learning is unilateral, but that transcriptional changes may be generalized to the untrained side at the memory maintenance stage. In such a case, transcriptional changes would not be detected using trained - untrained comparison. Dissociation of unilateral behavioral effects and bilateral structural changes have been reported previously (Wainwright et al., 2004, PMID: 15190093). A discussion of this possibility would be helpful.

3. The authors should also discuss the possibility that the Aplysia Tellabs Array, which is estimated to cover 50-60% of all transcripts expressed in the nervous system, might not detect some maintenance-related transcripts.

4. On page 11, the authors say "...none of the 1,253 transcripts regulated 1 day after training still showed clear regulation 5 days after training. "That statement appears to be at odds with the next section (page 13) where the authors discuss four specific transcripts that were upregulated at 5 days. Either clarify that those four transcripts were not regulated at 1 day, or change the statement of page 11 to "...none of the 1253 transcripts regulated 1 day after training, except for the four discussed in the next section, still showed clear regulation 5 days after training."

5. A significant number of animals (44 out of 77) were excluded due to weak training responses, bilateral expression, or other empirical factors. If 44 animals were excluded and 32 were used for the experiments, one animal is left unaccounted for.

6. Only one intermediate time point (5 day after training) was included in the study. The authors should discuss the possibility that this one time point might not be sufficient to capture a critical time window associated with forgetting and memory maintenance. Additional intermediate time points (e.g., 2 and 4 days after training) would solidify the conclusions.

7. Line 59, remove "to".

8. Line 61, write as "transcriptional changes decay within 1 week."

9. Line 161, correct to "expressed".

10. Line 221, write as "no remaining sensitization".

11. Figure 1, T is missing from 'Long-erm'

12. Figure 1, line 382. The "tail (arrows)" are not evident in the illustration.

13. Figure 2, line 391. The untrained line is blue, not black as stated.

14. Figure 2 legend, change "Memory" to "memory", change (black) to (blue), remove "in animals" from the last line.

15. Figure 3 legend, remove "data" from line 400, add the confidence interval shading to the figure, change "but each line" to "but with each line".

16. Figure 4 legend, change "but each line" to "but with each line".

17. Figure 5 legend. Be sure to specify the sources for the 1-day, 5-day, 7-day, and 8-day data. It appears that the 1- and 5-day data are replotted from Fig. 3, and the 7- and 8-day data are from either 1 or 2 previous papers by the authors. Please clarify and provide appropriate citations in the figure legend.

18. Figure 5 legend, change (black) to (blue).

19. The manuscript needs to be carefully proofread for grammatical errors.

**Reviewer #2**

The study represents an important continuation of a set of experiments to characterize transcriptional changes in a well-characterized memory circuit. The results show extraordinary robustness and very stringent controls increasing the confidence in the results. I have only a few additional conceptual caveats that I think need to be brough up in the discussion of this work.

Caveats.

Results are from 1 day of training that leads to memory that lasts for approximately 7 days. Training on additional days leads to longer lasting memory associated with the formation of new synapses. (Wainwright et al, 2002; doi: 10.1523/JNEUROSCI.22-10-04132.2002. (It may be that the longer-lasting memory is the one that requires more long-lasting changes in transcription and this is only induced with multiple days of training.

Transcription is only measured in the presynaptic neurons in this example, but gene expression changes may be required in both pre and post-synaptic neurons doi: 10.1523/JNEUROSCI.3284-14.2015. While it is not clear where long-lasting transcriptional changes are necessary, it is certainly conceivable that long-lasting transcriptional changes are more important in the post-synaptic neuron than in the pre-synaptic neuron. There are no direct connections between the pleural sensory neurons and the siphon withdrawal motor neurons. Furthermore, the important interneurons mediating this transfer of information has not been firmly established. This may complicate the question of where long-lasting gene expression is required in this system. These issues need to be acknowledged in the discussion.

## Author Response

Synthesis The study addresses the critical question on the relationship between gene transcription and memory retention using Aplysia as the model organism. The experimental design is rigorous, involving powerful within-subjects controls and high-stringency statistical analyses. The primary finding of the study is that most transcriptional changes decay more rapidly than memory expression. These observations imply that robust gene transcription might not be essential for maintenance of memory. Both reviewers agree on the rigor associated with the experimental design as well as on the importance of this study in unraveling the mechanistic basis of memory storage.

However, both reviewers underscore the need to place the findings of the study in context and acknowledge possible caveats within an expanded discussion section. In an expanded discussion section, please elaborate on possible alternate explanations for the findings here, covering both methodological and conceptual aspects. Please respond to all points raised by both reviewers, with appropriate changes made in the revised manuscript addressing their comments.

Reviewer's comments Reviewer #1 The manuscript presents a well-designed microarray analysis of transcriptional changes associated with long-term sensitization in Aplysia. The study finds that transcription fully decays at an intermediate behavioral stage (5 days post-sensitization training), a time-point when memory strength has weakened but is still maintained. The conclusion is that most transcriptional changes decay more rapidly than memory expression. This is an extremely interesting finding, suggesting that robust gene transcription may not be needed for the maintenance of memory. The study used a powerful within-subjects design, used unilateral sensitization in Aplysia to compare trained and untrained sides within the same animal, and used high-stringency statistical analyses. The manuscript could be improved by addressing the following concerns.

1. A novel finding is that memory expression persists at day 5, whereas nearly all transcriptional changes return to baseline. The authors need to discuss in more depth possible alternative mechanisms for this negative finding. For example, the negative results could be due, in part, to the use of whole ganglia for the transcriptional analysis. The use of whole ganglia might not capture some changes in expression, because changes from the specific subset of neurons actually encoding the memory may be diluted. The authors briefly discussed this possibility as a "refinement" process. They should provide additional discussion of this methodological limitation and implications of using whole ganglia for the analyses.

We agree. We had discussed the fact that cell-type specificity could cloud our results, and the need to conduct further work with better resolution. We have extended this discussion, adding a paragraph specifically touching on technical limitations of our approach, specifically addressing in more detail the drawbacks of analyzing transcription at the level of whole ganglia.

2. Another possible explanation for the negative results is the approach of comparing gene expressions in the pleural ganglia collected from the trained vs. untrained sides in the same animal. The authors should discuss the possibility that behavioral learning is unilateral, but that transcriptional changes may be generalized to the untrained side at the memory maintenance stage. In such a case, transcriptional changes would not be detected using trained - untrained comparison. Dissociation of unilateral behavioral effects and bilateral structural changes have been reported previously (Wainwright et al., 2004, PMID: 15190093). A discussion of this possibility would be helpful.

This is an important consideration, and we have now addressed this directly. While we cannot rule out changes in gene expression on the contralateral side, we do screen out animals that show behavioral changes on the untrained side. Furthermore, we do not believe that changes on the untrained side could fully explain the lack of transcriptional changes we observe 5 days after training, especially given that we were able to detect widespread changes at an earlier time point with the same approach and 1/2 the sample size. That is, for changes at 5 days but not 1 day to be cancelled out there would have to be late-breaking changes on the untrained side that match the changes on the trained side well enough to normalize them but yet without any behavioral consequence. We cannot rule out this possibility, but we do not think it is likely enough to strongly undermine our conclusion that most early transcriptional changes fade, at least at the level of whole ganglia.

3. The authors should also discuss the possibility that the Aplysia Tellabs Array, which is estimated to cover 50-60% of all transcripts expressed in the nervous system, might not detect some maintenance-related transcripts.

We agree, this is also an important consideration and have added discussion of this point. Again, though, we do not think this adequately explains our results. Although our microarray likely misses 40-50% of neuronally-expressed transcripts, we do detect widespread changes at 1 day. It seems unlikely that all subsequent changes in gene expression then shift exclusively to transcripts not represented on the array.

4. On page 11, the authors say "...none of the 1,253 transcripts regulated 1 day after training still showed clear regulation 5 days after training. "That statement appears to be at odds with the next section (page 13) where the authors discuss four specific transcripts that were upregulated at 5 days. Either clarify that those four transcripts were not regulated at 1 day, or change the statement of page 11 to "...none of the 1253 transcripts regulated 1 day after training, except for the four discussed in the next section, still showed clear regulation 5 days after training." Clarified. We started with transcripts regulated at 1 day excluding the small set we found to be very persistently regulared, leaving that set to be analyzed on their own in the next section. This is now made more clear.

5. A significant number of animals (44 out of 77) were excluded due to weak training responses, bilateral expression, or other empirical factors. If 44 animals were excluded and 32 were used for the experiments, one animal is left unaccounted for.

We fixed this typo. There were 32 animals discarded due to protocol error: 3 due to a shock applied to the wrong side; 29 due to uneven or poor RNA isolation. We had the specific counts, but mis-reported this total as 31.

6. Only one intermediate time point (5 day after training) was included in the study. The authors should discuss the possibility that this one time point might not be sufficient to capture a critical time window associated with forgetting and memory maintenance. Additional intermediate time points (e.g., 2 and 4 days after training) would solidify the conclusions.

This is true. We have noted in the discussion the importance of conducting a detailed time-course study.

7. Line 59, remove "to".

Fixed 8. Line 61, write as "transcriptional changes decay within 1 week." Fixed 9. Line 161, correct to "expressed".

Fixed 10. Line 221, write as "no remaining sensitization".

Fixed 11. Figure 1, T is missing from 'Long-erm' Fixed 12. Figure 1, line 382. The "tail (arrows)" are not evident in the illustration.

Fixed 13. Figure 2, line 391. The untrained line is blue, not black as stated.

Fixed 14. Figure 2 legend, change "Memory" to "memory", change (black) to (blue), remove "in animals" from the last line.

Fixed 15. Figure 3 legend, remove "data" from line 400, add the confidence interval shading to the figure, change "but each line" to "but with each line".

Fixed. The Confidence interval of the line of best fit is shaded in gray, but is too narrow to be visible; this is now noted in the legend.

16. Figure 4 legend, change "but each line" to "but with each line".

Fixed 17. Figure 5 legend. Be sure to specify the sources for the 1-day, 5-day, 7-day, and 8-day data. It appears that the 1- and 5-day data are replotted from Fig. 3, and the 7- and 8-day data are from either 1 or 2 previous papers by the authors. Please clarify and provide appropriate citations in the figure legend.

These had been removed for blind review; all sources are now in the figure legend 18. Figure 5 legend, change (black) to (blue).

Fixed 19. The manuscript needs to be carefully proofread for grammatical errors.

Done Reviewer #2 The study represents an important continuation of a set of experiments to characterize transcriptional changes in a well-characterized memory circuit. The results show extraordinary robustness and very stringent controls increasing the confidence in the results. I have only a few additional conceptual caveats that I think need to be brought up in the discussion of this work.

Caveats.

Results are from 1 day of training that leads to memory that lasts for approximately 7 days. Training on additional days leads to longer lasting memory associated with the formation of new synapses. (Wainwright et al, 2002; doi: 10.1523/JNEUROSCI.22-10-04132.2002. (It may be that the longer-lasting memory is the one that requires more long-lasting changes in transcription and this is only induced with multiple days of training.

We agree. We note in our discussion that it will be important to try more intensive training protocols that will enable a test of transcription during maintenance that is not clouded by partial forgetting.

Transcription is only measured in the presynaptic neurons in this example, but gene expression changes may be required in both pre and post-synaptic neurons doi: 10.1523/JNEUROSCI.3284-14.2015. While it is not clear where long-lasting transcriptional changes are necessary, it is certainly conceivable that long-lasting transcriptional changes are more important in the post-synaptic neuron than in the pre-synaptic neuron. There are no direct connections between the pleural sensory neurons and the siphon withdrawal motor neurons. Furthermore, the important interneurons mediating this transfer of information has not been firmly established. This may complicate the question of where long This is a good point. We have expanded our discussion of refinement of transcription to specifically discuss the fact that it could change the loci of storage, moving ongoing transcriptional changes from the pleural ganglia we measured to the pedal ganglia or peripheral nervous system.
